# Kyzatrex - Oral testosterone replacement therapy

**DOI:** 10.1016/j.amsu.2022.104625

**Published:** 2022-09-11

**Authors:** Adam Ali Asghar, Mahnoor Rehan Hashmi, Rushaan Ahmed, Yumna Khabir

**Affiliations:** Department of Medicine, Dow University of Health Sciences, Mission Rd, New Labour Colony Nanakwara, Karachi, Sindh, Pakistan

Male hypogonadism, characterized by the body's inability to secrete sufficient amounts of testosterone [1], affects approximately 40% of men over the age of 45 globally [1]. It is commonly present in patients with Type 2 Diabetes Mellitus and Non-alcoholic Steatohepatitis (NASH) [1]. Symptoms of hypogonadism include lack of energy, loss of muscle mass, weight gain, decreased libido, cognitive decline, sleep disturbances and other mood disorders such as depression and anxiety [1]. It may be due to a primary disease or secondary to a defect in the hypothalamic-pituitary axis, each of which may be primary (testicular or hypergonadotropic) or secondary (central or hypogonadotropic) hypogonadism [2]. With respect to the primarily affected testicular compartment, hypogonadism can present with complete hypogonadism or it can be due to dissociated cell-specific hypogonadism, in which there is disruption of one of the cell populations (Sertoli, Leydig or germ cells) while the function of others remains intact [2].

The standard treatment strategy available for male hypogonadism is Testosterone replacement therapy (TRT) [3]. It is available in different formulations such as transdermal patches, intramuscular injections, subcutaneous pellets, nasal gels or capsules [3]. Kyzatrex, a type of oral TRT has been approved by the Food and Drug Administration (FDA) on 2nd August 2022 [1]. This oral soft gel capsule consist of testosterone undecanoate (TU) dissolved in a combination of lipids (long chain fatty acids such as oleic acid) and other solubilizers like borage seed oil having C-20 fatty acids, peppermint oil and a hydrophilic surfactant such as castor oil [4]. This formulation helps dissolution of highly lipophilic molecules of TU for rapid absorption in the blood along with food [4]. It is a huge milestone as it brings a solution to complications of previous administration methods like application site reactions induced by intramuscular injections, gels and patches [1]. Systemic delivery of oral TU occurs instantly (>97%) via the intestinal lymphatic system thus bypassing the liver completely. Once in the circulation testosterone is freed from TU by the action of endogenous non-specific esterases [4]. The undecanoic side chain (a C-11 fatty acid) is pharmacologically inert and is mobilized by β-oxidation to acetyl coenzyme A (CoA) [4]. Swedloff et al. determined the efficacy and safety of oral testosterone undecanoate in a total of 166 patients [4]. The study concluded that in 87.3% patients, oral TU restored normal level of serum testosterone [4]. Kyzatrex has been approved in 3 dosage strengths; 100 mg, 150 mg and 200 mg [1]. Some common adverse effects observed were increased blood pressure which may lead to heart attack/stroke, increase in red blood cell count (hematocrit) or hemoglobin increasing the risk of blood clots in legs or lungs, increased risk of prostate cancer, liver problems, edema in ankles and feet, enlarged, painful breasts and sleep apnea [1]. It is not recommended to use if you have breast cancer, prostate cancer, pregnancy or have low testosterone without any medical condition.^1^

Managing hyperprolactinemia, obesity and chronic diseases like type 2 diabetes may also help regulate testosterone levels along with TRT [3]. Lifestyle modifications like maintaining healthy weight and increasing physical activity have also contributed to improving testosterone levels [3]. TRT inhibits spermatogenesis, and thus, cannot be suggested to patients with a desire to have children in the future [3]. In such cases, gonadotropins come to the rescue as they are capable of inducing spermatogenesis in 75% of patients according to a study [3]. More extensive studies are required to determine if Kyzatrex could be given along gonadotropins or if there are any other underlying conditions that should be investigated before prescribing tis oral form of TRT.

## Ethical approval

The approval was not needed.

## Source of funding

The authors have no financial or proprietary interest in the subject matter of this article.

## Author contribution

Adam Ali Asghar; Conceptualization, literature review and manuscript writing. Mahnoor Rehan Hashmi; Literature review and manuscript writing. Rushaan Ahmed; Literature review and manuscript writing. Yumna Khabir; Literature review and manuscript writing.

## Trail registry number

None.

## Guarantor

Adam Ali Asghar, Mahnoor Rehan Hashmi, Rushaan Ahmed, Yumna Khabir.

## Declaration of competing interest

The authors have no conflict of interest.
